# The monoclinic form of trilithium dichromium(III) tris­(orthophosphate)

**DOI:** 10.1107/S1600536813026433

**Published:** 2013-09-28

**Authors:** Joobin Sun, Pilsoo Kim, Hoseop Yun

**Affiliations:** a Department of Energy Systems Research and Department of Chemistry, Ajou University, Suwon 443-749, Republic of Korea

## Abstract

The monoclinic form of trilithium dichromium(III) tris­(ortho­phosphate), Li_3_Cr_2_(PO_4_)_3_, was prepared by the reactive halide flux method. The structure of the title compound is composed of a three-dimensional anionic framework with composition _∞_^3^[Cr_2_(PO_4_)_3_]^3−^ and Li^+^ ions situated in the empty channels. The rigid framework built up from CrO_6_ octa­hedra and PO_4_ tetra­hedra is the same as that found in other monoclinic Li_3_ *M*_2_(PO_4_)_3_ (*M* = Fe, Sc, V) phases. The three Li^+^ cations of Li_3_Cr_2_(PO_4_)_3_ are unequally disordered over six crystallographically different sites. The classical charge balance of the title compound can be represented as [Li^+^]_3_[Cr^3+^]_2_[P^5+^]_3_[O^2−^]_12_. Solid-state UV/Vis spectra indicate that the crystal filed splitting (Δ_0_) of the Cr^3+^ ion is around 2.22 eV.

## Related literature

For the structures of Li_3_ *M*_2_(PO_4_)_3_ (*M* = Fe, Sc, Cr, V), see: d’Yvoire *et al.* (1983 ▶); Verin *et al.* (1985 ▶); Maksimov *et al.* (1986 ▶). The structures of the ortho­rhom­bic form of Li_3_Cr_2_(PO_4_)_3_ have been investigated by Genkina *et al.* (1991 ▶). Structural studies of Li_3_V_2_(PO_4_)_3_ based on single-crystal data have been reported previously by Kee & Yun (2013 ▶). The general structural features of the monoclinic phases have been discussed by Patoux *et al.* (2003 ▶); Fu *et al.* (2010 ▶); Yang *et al.* (2010 ▶). For ionic radii, see: Shannon (1976 ▶).

## Experimental

### 

#### Crystal data

Li_3_Cr_2_(PO_4_)_3_
*M* *_r_* = 409.73
Monoclinic, 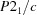

*a* = 8.4625 (4) Å
*b* = 8.5560 (3) Å
*c* = 14.5344 (5) Å
β = 125.186 (2)°
*V* = 860.08 (6) Å^3^
*Z* = 4
Mo *K*α radiation
μ = 3.16 mm^−1^
*T* = 290 K
0.36 × 0.12 × 0.10 mm

#### Data collection

Rigaku R-AXIS RAPID S diffractometer
Absorption correction: multi-scan (*ABSCOR*; Higashi, 1995 ▶) *T*_min_ = 0.688, *T*_max_ = 1.000
8163 measured reflections
1962 independent reflections
1887 reflections with *I* > 2σ(*I*)
*R*_int_ = 0.021

#### Refinement

*R*[*F* ^2^ > 2σ(*F* ^2^)] = 0.026
*wR*(*F* ^2^) = 0.068
*S* = 1.14
1962 reflections
184 parameters
1 restraint
Δρ_max_ = 0.71 e Å^−3^
Δρ_min_ = −0.71 e Å^−3^

### 

Data collection: *RAPID-AUTO* (Rigaku, 2006 ▶); cell refinement: *RAPID-AUTO*; data reduction: *RAPID-AUTO*; program(s) used to solve structure: *SHELXS97* (Sheldrick, 2008 ▶); program(s) used to refine structure: *SHELXL97* (Sheldrick, 2008 ▶); molecular graphics: *DIAMOND* (Brandenburg, 1999 ▶); software used to prepare material for publication: *WinGX* (Farrugia, 2012 ▶) and *publCIF* (Westrip, 2010 ▶).

## Supplementary Material

Crystal structure: contains datablock(s) I, New_Global_Publ_Block. DOI: 10.1107/S1600536813026433/wm2772sup1.cif

Structure factors: contains datablock(s) I. DOI: 10.1107/S1600536813026433/wm2772Isup2.hkl

Additional supplementary materials:  crystallographic information; 3D view; checkCIF report

## Figures and Tables

**Fig. 1.**
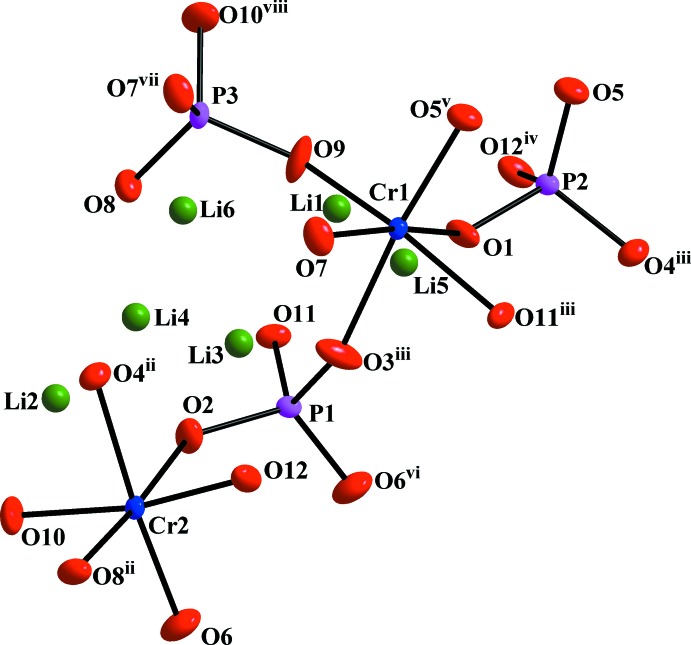
A view showing the local coordination environments of Cr and P atoms with the atom labeling scheme. Displacement ellipsoids for Cr, P, and O atoms are drawn at the 70% probability level. [Symmetry codes: (ii) -x, y+1/2, -z+1/2; (iii) -x+1, y+1/2, -z+1/2; (iv) -x+1, y-1/2, -z+1/2; (v) -x+1, -y+1, -z; (vi) -x+1, -y+1, -z+1; (vii) -x, -y+1, -z; (viii) x, -y+1/2, z-1/2. ].

**Fig. 2.**
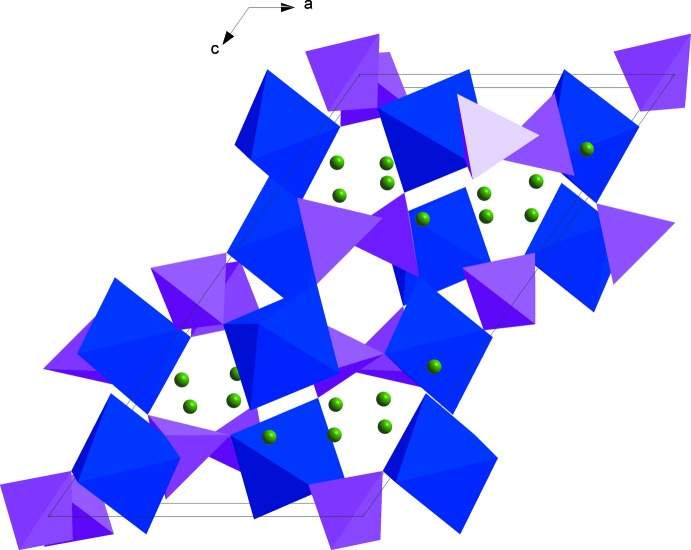
The polyhedral representation of the anionic framework structure built up from [Cr2(PO4)3] units. The disordered Li+ cations are located in the channels of this framework.

## supplementary crystallographic information

### 1. Comment

The structures of trilithium dimetal tris(orthophosphates), Li_3_*M*_2_(PO_4_)_3_ (*M* = Fe, Sc, Cr, V) have been widely investigated due to their ion transport properties and temperature-dependent phase transitions (d'Yvoire *et al.*, 1983; Verin *et al.*, 1985; Maksimov *et al.*, 1986). For Li_3_Cr_2_(PO_4_)_3_, the structure of the orthorhombic phase has been studied based on single-crystal diffraction data (Genkina *et al.*, 1991). The monoclinic and rhombohedral phases have been identified by powder diffraction techniques but no detailed structure determinations have been reported yet (d'Yvoire *et al.*, 1983). In attempts to prepare new mixed alkali metal phosphates by using various alkali metal halides, the monoclinic form of Li_3_Cr_2_(PO_4_)_3_ has been isolated as single crystals and the detailed structural characterization of this phase is reported here.

The anionic framework of Li_3_Cr_2_(PO_4_)_3_ is the same as that of the previously reported monoclinic Li_3_V_2_(PO_4_)_3_ structure (Kee & Yun, 2013). The general structural features of this phase have been discussed previously (Patoux *et al.*, 2003; Fu *et al.*, 2010). Figure 1 shows the coordination environment of the Cr and P atoms. CrO_6_ octahedra are joined to PO_4_ tetrahedra forming a [Cr_2_(PO_4_)_3_] unit. These units share a terminal oxygen atom to construct the anionic three-dimensional framework, ^3^_∞_[Cr_2_(PO_4_)_3_]^3-^ (Fig. 2). The Cr—O distances (1.9007 (18)–2.0392 (18) Å) are in good agreement with those calculated from their ionic radii (2.00 Å; Shannon, 1976), assuming a valence of +III for Cr.

The Li^+^ ions in the empty channels are surrounded by four O atoms in distorted tetrahedral coordinations. There are six crystallographically independent Li sites for this phase and three Li^+^ ions are unequally disordered over them. It has been reported that the positions of Li^+^ ions in Li_3_V_2_(PO_4_)_3_ can vary depending on the synthetic conditions while those of the V, P, and O atoms comprising the rigid framework remain intact (Yang *et al.*, 2010). The positions of the Li1, Li3, and Li4 sites in this work are very close to those of the ordered Li sites found in Li_3_V_2_(PO_4_)_3_ (Kee & Yun, 2013).

The classical charge balance of the title compound can be represented as [Li^+^]_3_[Cr^3+^]_2_[P^5+^]_3_[O^2-^]_12_. Solid-state UV/Vis spectra indicate that the crystal filed splitting(Δ_0_) of the Cr^3+^ ion is around 2.22 eV, which is in agreement with the green color of the crystals.

### 2. Experimental

The title compound, Li_3_Cr_2_(PO_4_)_3_, was prepared by the reaction of the elements with the use of the reactive halide-flux technique. A combination of the pure elements, Cr powder (Cerac, 99.95%) and P powder (Aldrich, 99%), were mixed in a fused silica tube in a molar ratio of Cr:P = 1:1 and then LiCl (Cerac, 99.8%) and CsCl (Alfa, 99.9%) mixed in molar ratio of LiCl:CsCl = 4:1 were added. The mass ratio of the reactants and the halides was 1:3. The tube was evacuated to 0.133 Pa, sealed, and heated gradually (30 K/h) to 1123 K, where it was kept for 48 h. The tube was cooled to room temperature at a rate of 4 K/h. The excess halide was removed with water and greenish block-shaped crystals were obtained. The crystals are stable in air and water. A qualitative X-ray fluorescence analysis of selected crystals indicated the presence of Cr and P. The final composition of the compound was determined by single-crystal X-ray diffraction.

### 3. Refinement

After the positions of heavy elements (Cr, P, and O) had been established, six significant residual peaks suitable for Li^+^ sites were revealed by difference Fourier maps. A model including disorder for these sites was applied. The sum of the Li^+^ occupancies was fixed to 3 to meet the charge balance of the compound; the temperature factors of all Li sites were refined isotropically. The highest peak (0.71 e Å^-3^) and the deepest hole (-0.49 e Å^-3^) are 0.57 Å and 0.81 Å from atom Li4 and Cr1, respectively.

### Crystal data

**Table:**

Li_3_Cr_2_(PO_4_)_3_	*F*(000) = 792
*M**_r_* = 409.73	*D*_x_ = 3.164 Mg m^−^^3^
Monoclinic, *P*2_1_/*c*	Mo *K*α radiation, λ = 0.71073 Å
Hall symbol: -P 2ybc	Cell parameters from 8071 reflections
*a* = 8.4625 (4) Å	θ = 3.4–27.7°
*b* = 8.5560 (3) Å	µ = 3.16 mm^−^^1^
*c* = 14.5344 (5) Å	*T* = 290 K
β = 125.186 (2)°	Block, green
*V* = 860.08 (6) Å^3^	0.36 × 0.12 × 0.10 mm
*Z* = 4	

### Data collection

**Table:**

Rigaku R-AXIS RAPID S diffractometer	1962 independent reflections
Radiation source: Sealed X-ray tube	1887 reflections with *I* > 2σ(*I*)
Graphite monochromator	*R*_int_ = 0.021
ω scans	θ_max_ = 27.5°, θ_min_ = 3.4°
Absorption correction: multi-scan (*ABSCOR*; Higashi, 1995)	*h* = −10→10
*T*_min_ = 0.688, *T*_max_ = 1.000	*k* = −11→10
8163 measured reflections	*l* = −18→18

### Refinement

**Table:**

Refinement on *F*^2^	1 restraint
Least-squares matrix: full	Primary atom site location: structure-invariant direct methods
*R*[*F*^2^ > 2σ(*F*^2^)] = 0.026	Secondary atom site location: difference Fourier map
*wR*(*F*^2^) = 0.068	*w* = 1/[σ^2^(*F*_o_^2^) + (0.0319*P*)^2^ + 1.8772*P*] where *P* = (*F*_o_^2^ + 2*F*_c_^2^)/3
*S* = 1.14	(Δ/σ)_max_ < 0.001
1962 reflections	Δρ_max_ = 0.71 e Å^−^^3^
184 parameters	Δρ_min_ = −0.71 e Å^−^^3^

### Special details

**Table:**

Geometry. All e.s.d.'s (except the e.s.d. in the dihedral angle between two l.s. planes) are estimated using the full covariance matrix. The cell e.s.d.'s are taken into account individually in the estimation of e.s.d.'s in distances, angles and torsion angles; correlations between e.s.d.'s in cell parameters are only used when they are defined by crystal symmetry. An approximate (isotropic) treatment of cell e.s.d.'s is used for estimating e.s.d.'s involving l.s. planes.

### Fractional atomic coordinates and isotropic or equivalent isotropic displacement parameters (Å^2^)

**Table:**

	*x*	*y*	*z*	*U*_iso_*/*U*_eq_	Occ. (<1)
Li1	0.4750 (10)	0.2119 (8)	0.1752 (6)	0.022 (2)*	0.71 (2)
Li2	0.104 (2)	0.208 (2)	0.3364 (14)	0.022 (6)*	0.30 (2)
Li3	0.1164 (11)	0.5885 (9)	0.1920 (7)	0.017 (3)*	0.59 (2)
Li4	0.1935 (14)	0.1892 (12)	0.2627 (8)	0.036 (3)*	0.64 (3)
Li5	0.672 (3)	0.227 (2)	0.2586 (15)	0.019 (6)*	0.27 (2)
Li6	0.273 (3)	0.059 (3)	0.1835 (19)	0.072 (8)*	0.48 (3)
Cr1	0.36305 (5)	0.53352 (4)	0.11142 (3)	0.00608 (11)	
Cr2	0.13604 (5)	0.53218 (5)	0.38801 (3)	0.00787 (11)	
P1	0.46068 (8)	0.39077 (7)	0.35382 (5)	0.00707 (13)	
P2	0.75261 (8)	0.38724 (7)	0.14717 (5)	0.00693 (13)	
P3	0.04107 (8)	0.25059 (7)	0.00513 (5)	0.00717 (13)	
O1	0.6057 (2)	0.4162 (2)	0.17588 (15)	0.0129 (4)	
O2	0.2909 (3)	0.3809 (2)	0.36484 (16)	0.0138 (4)	
O3	0.5955 (3)	0.0119 (2)	0.23933 (16)	0.0181 (4)	
O4	0.0766 (3)	0.0017 (2)	0.27885 (15)	0.0124 (3)	
O5	0.6674 (3)	0.4170 (2)	0.02547 (15)	0.0135 (4)	
O6	0.3515 (3)	0.5558 (2)	0.54233 (16)	0.0197 (4)	
O7	0.1224 (3)	0.6330 (2)	0.06579 (16)	0.0135 (4)	
O8	0.0340 (2)	0.1757 (2)	0.09856 (15)	0.0135 (4)	
O9	0.2391 (2)	0.3295 (2)	0.06304 (17)	0.0166 (4)	
O10	0.0217 (3)	0.3652 (2)	0.41922 (16)	0.0156 (4)	
O11	0.4784 (2)	0.2282 (2)	0.31441 (14)	0.0106 (3)	
O12	0.1852 (3)	0.7155 (2)	0.32091 (15)	0.0140 (4)	

### Atomic displacement parameters (Å^2^)

**Table:**

	*U*^11^	*U*^22^	*U*^33^	*U*^12^	*U*^13^	*U*^23^
Cr1	0.00427 (18)	0.00702 (19)	0.00645 (19)	0.00016 (12)	0.00280 (15)	0.00019 (13)
Cr2	0.00503 (18)	0.0106 (2)	0.00828 (19)	−0.00115 (13)	0.00402 (15)	−0.00151 (14)
P1	0.0064 (3)	0.0078 (3)	0.0061 (3)	0.0019 (2)	0.0030 (2)	0.0007 (2)
P2	0.0054 (3)	0.0085 (3)	0.0063 (3)	0.0014 (2)	0.0031 (2)	0.0000 (2)
P3	0.0053 (3)	0.0063 (3)	0.0100 (3)	−0.0001 (2)	0.0045 (2)	−0.0001 (2)
O1	0.0089 (8)	0.0216 (9)	0.0095 (8)	0.0060 (7)	0.0060 (7)	0.0022 (7)
O2	0.0112 (8)	0.0133 (8)	0.0197 (9)	−0.0001 (7)	0.0106 (7)	−0.0023 (7)
O3	0.0348 (11)	0.0105 (8)	0.0154 (9)	−0.0066 (8)	0.0182 (9)	−0.0043 (7)
O4	0.0105 (8)	0.0128 (8)	0.0095 (8)	0.0013 (7)	0.0033 (7)	0.0010 (7)
O5	0.0140 (8)	0.0171 (9)	0.0089 (8)	0.0027 (7)	0.0062 (7)	0.0018 (7)
O6	0.0117 (9)	0.0235 (10)	0.0146 (9)	−0.0025 (8)	0.0023 (8)	−0.0065 (8)
O7	0.0117 (8)	0.0122 (8)	0.0186 (9)	0.0053 (7)	0.0099 (7)	0.0053 (7)
O8	0.0100 (8)	0.0180 (9)	0.0108 (8)	−0.0009 (7)	0.0050 (7)	0.0038 (7)
O9	0.0076 (8)	0.0083 (8)	0.0302 (11)	−0.0018 (7)	0.0087 (8)	−0.0017 (8)
O10	0.0115 (8)	0.0191 (9)	0.0157 (9)	−0.0037 (7)	0.0075 (7)	0.0043 (7)
O11	0.0125 (8)	0.0081 (8)	0.0086 (8)	0.0038 (6)	0.0045 (7)	0.0002 (6)
O12	0.0216 (9)	0.0100 (8)	0.0151 (8)	−0.0057 (7)	0.0133 (8)	−0.0041 (7)

### Geometric parameters (Å, º)

**Table:**

Li1—O3	1.934 (7)	Cr1—O5^v^	1.9007 (18)
Li1—O9	1.977 (7)	Cr1—O7	1.9380 (17)
Li1—O11	2.011 (7)	Cr1—O9	1.9471 (18)
Li1—O1	2.066 (7)	Cr1—O1	1.9709 (17)
Li2—O4	1.908 (17)	Cr1—O3^iii^	1.9965 (18)
Li2—O2	2.028 (17)	Cr1—O11^iii^	2.0172 (17)
Li2—O10	2.170 (17)	Cr2—O6	1.9192 (19)
Li2—O12^i^	2.184 (17)	Cr2—O10	1.9208 (18)
Li3—O7	1.902 (8)	Cr2—O8^ii^	1.9847 (18)
Li3—O12	1.943 (8)	Cr2—O2	2.0041 (18)
Li3—O4^ii^	2.049 (8)	Cr2—O12	2.0129 (18)
Li3—O3^iii^	2.137 (8)	Cr2—O4^ii^	2.0392 (18)
Li4—O8	1.953 (10)	P1—O6^vi^	1.4994 (19)
Li4—O4	1.969 (10)	P1—O2	1.5368 (18)
Li4—O2	2.040 (10)	P1—O11	1.5443 (17)
Li4—O11	2.098 (10)	P1—O3^iii^	1.5463 (19)
Li5—O1	1.900 (18)	P2—O5	1.4979 (18)
Li5—O3	1.916 (18)	P2—O12^iv^	1.5394 (18)
Li5—O12^iv^	2.103 (18)	P2—O1	1.5424 (17)
Li5—O11	2.206 (18)	P2—O4^iii^	1.5532 (18)
Li5—O7^iv^	2.252 (18)	P3—O7^vii^	1.5248 (18)
Li6—O8	1.93 (2)	P3—O10^viii^	1.5268 (19)
Li6—O1^iv^	2.08 (2)	P3—O9	1.5319 (18)
Li6—O11	2.22 (2)	P3—O8	1.5337 (18)
Li6—O3	2.39 (2)		
			
O5^v^—Cr1—O7	93.57 (8)	O2—Cr2—O12	94.84 (8)
O5^v^—Cr1—O9	95.83 (9)	O6—Cr2—O4^ii^	175.15 (8)
O7—Cr1—O9	91.63 (8)	O10—Cr2—O4^ii^	88.07 (8)
O5^v^—Cr1—O1	94.97 (8)	O8^ii^—Cr2—O4^ii^	90.18 (7)
O7—Cr1—O1	171.08 (8)	O2—Cr2—O4^ii^	86.09 (8)
O9—Cr1—O1	84.97 (8)	O12—Cr2—O4^ii^	79.09 (8)
O5^v^—Cr1—O3^iii^	172.27 (8)	O6^vi^—P1—O2	115.09 (11)
O7—Cr1—O3^iii^	84.53 (8)	O6^vi^—P1—O11	112.02 (11)
O9—Cr1—O3^iii^	91.72 (9)	O2—P1—O11	106.60 (10)
O1—Cr1—O3^iii^	87.34 (8)	O6^vi^—P1—O3^iii^	106.84 (12)
O5^v^—Cr1—O11^iii^	91.32 (8)	O2—P1—O3^iii^	107.11 (11)
O7—Cr1—O11^iii^	93.70 (8)	O11—P1—O3^iii^	108.98 (10)
O9—Cr1—O11^iii^	170.80 (8)	O5—P2—O12^iv^	111.51 (10)
O1—Cr1—O11^iii^	88.66 (8)	O5—P2—O1	112.18 (10)
O3^iii^—Cr1—O11^iii^	81.34 (8)	O12^iv^—P2—O1	105.13 (10)
O6—Cr2—O10	94.07 (9)	O5—P2—O4^iii^	109.45 (11)
O6—Cr2—O8^ii^	94.27 (8)	O12^iv^—P2—O4^iii^	111.89 (10)
O10—Cr2—O8^ii^	86.83 (8)	O1—P2—O4^iii^	106.55 (10)
O6—Cr2—O2	89.50 (8)	O7^vii^—P3—O10^viii^	104.06 (10)
O10—Cr2—O2	91.50 (8)	O7^vii^—P3—O9	111.22 (10)
O8^ii^—Cr2—O2	175.97 (8)	O10^viii^—P3—O9	107.65 (11)
O6—Cr2—O12	99.31 (8)	O7^vii^—P3—O8	112.88 (10)
O10—Cr2—O12	165.23 (8)	O10^viii^—P3—O8	114.36 (11)
O8^ii^—Cr2—O12	85.96 (8)	O9—P3—O8	106.63 (11)

Symmetry codes: (i) −*x*, *y*−1/2, −*z*+1/2; (ii) −*x*, *y*+1/2, −*z*+1/2; (iii) −*x*+1, *y*+1/2, −*z*+1/2; (iv) −*x*+1, *y*−1/2, −*z*+1/2; (v) −*x*+1, −*y*+1, −*z*; (vi) −*x*+1, −*y*+1, −*z*+1; (vii) −*x*, −*y*+1, −*z*; (viii) *x*, −*y*+1/2, *z*−1/2.
